# Supplementary material for: Outcomes of the KC life 360 intervention: Improving employment and housing for persons living with HIV
Source: PLoS One. 2022 Sep 16;17(9):e0274923. doi: 10.1371/journal.pone.0274923 (PMC9481028; doi:10.1371/journal.pone.0274923)
Supplement: S1 Table — (DOCX) [file pone.0274923.s002.docx]

| **Table 1. Results from Ordinal Logistic GEE for Housing.** | | | | | |
| --- | --- | --- | --- | --- | --- |
| Coefficient | Estimate | SE | Wald Z | *p* | OR |
| *Intercept Only Model* | | | | | |
| *j* *>* 1 | 1.003 | 0.190 | 27.747 | < .001 | 2.726 |
| *j* *>* 2 | 0.790 | 0.190 | 17.307 | < .001 | 2.203 |
| *j >* 3 | - 0.572 | 0.194 | 8.689 | 0.003 | 0.564 |
| *By Measurement Wave* | | | | | |
| *j* *>* 1 | - 4.885 | 1.339 | 13.316 | < .001 | 0.008 |
| *j* *>* 2 | - 5.090 | 1.369 | 13.818 | < .001 | 0.006 |
| *j >* 3 | - 7.394 | 1.498 | 24.360 | < .001 | 0.001 |
| Time | 3.158 | 0.877 | 12.967 | < .001 | 23.524 |
| *By Measurement Month* | | | | | |
| *j* *>* 1 | - 1.776 | 0.523 | 11.559 | < .001 | 0.169 |
| *j* *>* 2 | - 1.990 | 0.557 | 12.789 | < .001 | 0.137 |
| *j >* 3 | - 4.294 | 0.657 | 42.689 | < .001 | 0.014 |
| Time | 0.531 | 0.152 | 12.250 | < .001 | 1.701 |

Note: SE = Standard error, OR = Odds ratio.
