# Supplementary material for: Outcomes of the KC life 360 intervention: Improving employment and housing for persons living with HIV
Source: PLoS One. 2022 Sep 16;17(9):e0274923. doi: 10.1371/journal.pone.0274923 (PMC9481028; doi:10.1371/journal.pone.0274923)
Supplement: S2 Table — (DOCX) [file pone.0274923.s003.docx]

| **Table 2. Results from Ordinal Logistic GEE for Employment.** | | | | | |
| --- | --- | --- | --- | --- | --- |
| Coefficient | Estimate | SE | Wald Z | *p* | OR |
| *Intercept Only Model* | | | | | |
| *j* *>* 1 | - 0.630 | 0.152 | 17.153 | < .001 | 0.533 |
| *j* *>* 2 | - 0.741 | 0.155 | 22.903 | < .001 | 0.477 |
| *j >* 3 | - 0.929 | 0.157 | 35.083 | < .001 | 0.395 |
| *j > 4* | - 1.394 | 0.184 | 57.649 | < .001 | 0.248 |
| *By Measurement Wave* | | | | | |
| *j* *>* 1 | - 1.921 | 0.415 | 21.429 | < .001 | 0.146 |
| *j* *>* 2 | - 2.036 | 0.420 | 23.475 | < .001 | 0.131 |
| *j >* 3 | - 2.233 | 0.427 | 27.352 | < .001 | 0.107 |
| *j > 4* | - 2.728 | 0.444 | 37.763 | < .001 | 0.065 |
| Time | 0.659 | 0.195 | 11.380 | 0.001 | 1.933 |
| *By Measurement Month* | | | | | |
| *j* *>* 1 | - 1.335 | 0.263 | 25.657 | < .001 | 0.263 |
| *j* *>* 2 | - 1.449 | 0.269 | 29.063 | < .001 | 0.235 |
| *j >* 3 | - 1.645 | 0.275 | 35.854 | < .001 | 0.193 |
| *j > 4* | - 2.139 | 0.296 | 52.142 | < .001 | 0.118 |
| Time | 0.116 | 0.035 | 11.003 | 0.001 | 1.123 |

Note: SE = Standard error, OR = Odds ratio.
