# Supplementary material for: Outcomes of the KC life 360 intervention: Improving employment and housing for persons living with HIV
Source: PLoS One. 2022 Sep 16;17(9):e0274923. doi: 10.1371/journal.pone.0274923 (PMC9481028; doi:10.1371/journal.pone.0274923)
Supplement: S3 Table — (DOCX) [file pone.0274923.s004.docx]

| **Table 3. Results from Binary Logistic GEE for Employment.** | | | | | |
| --- | --- | --- | --- | --- | --- |
| Coefficient | Estimate | SE | Wald Z | *p* | OR |
| *Intercept Only Model* | | | | | |
| *j* *>* 0 | - 0.620 | 0.147 | 17.754 | < .001 | 0.538 |
| *By Measurement Wave* |  |  |  |  |  |
| *j* *>* 0 | - 1.495 | 0.376 | 15.775 | < .001 | 0.224 |
| Time | 0.464 | 0.181 | 6.598 | 0.010 | 1.590 |
| *By Measurement Month* |  |  |  |  |  |
| *j* *>* 0 | - 1.092 | 0.243 | 20.253 | < .001 | 0.336 |
| Time | 0.083 | 0.033 | 6.357 | 0.012 | 1.087 |

Note: SE = Standard error, OR = Odds ratio.
