# Supplementary material for: Outcomes of the KC life 360 intervention: Improving employment and housing for persons living with HIV
Source: PLoS One. 2022 Sep 16;17(9):e0274923. doi: 10.1371/journal.pone.0274923 (PMC9481028; doi:10.1371/journal.pone.0274923)
Supplement: S4 Table — (DOCX) [file pone.0274923.s005.docx]

| **Table 4. Results from Binary Logistic GEE for Viral Suppression.** | | | | | |
| --- | --- | --- | --- | --- | --- |
| Coefficient | Estimate | SE | Wald Z | *p* | OR |
| *Intercept Only Model* | | | | | |
| *j* *>* 0 | 1.206 | 0.166 | 52.618 | 0.000 | 3.340 |
| *By Measurement Wave* |  |  |  |  |  |
| *j* *>* 0 | 0.382 | 0.422 | 0.819 | 0.365 | 1.465 |
| Time | 0.468 | 0.233 | 4.036 | 0.045 | 1.597 |
| *By Measurement Month* |  |  |  |  |  |
| *j* *>* 0 | 0.790 | 0.257 | 9.438 | 0.002 | 2.203 |
| Time | 0.083 | 0.043 | 3.650 | 0.056 | 1.087 |

Note: SE = Standard error, OR = Odds ratio.
