# Supplementary material for: Outcomes of the KC life 360 intervention: Improving employment and housing for persons living with HIV
Source: PLoS One. 2022 Sep 16;17(9):e0274923. doi: 10.1371/journal.pone.0274923 (PMC9481028; doi:10.1371/journal.pone.0274923)
Supplement: S5 Table — (DOCX) [file pone.0274923.s006.docx]

| **Table 5****.** **Results from Binary Logistic GEE for Retention in Care.** | | | | | |
| --- | --- | --- | --- | --- | --- |
| Coefficient | Estimate | SE | Wald Z | *p* | OR |
| *Intercept Only Model* | | | | | |
| *j* *>* 0 | - 1.064 | 0.152 | 48.766 | 0.000 | 0.345 |
| *By Measurement Wave* |  |  |  |  |  |
| *j* *>* 0 | - 0.699 | 0.425 | 2.705 | 0.100 | 0.497 |
| Time | - 0.199 | 0.222 | 0.804 | 0.370 | 0.820 |
| *By Measurement Month* |  |  |  |  |  |
| *j* *>* 0 | - 0.905 | 0.262 | 11.975 | 0.001 | 0.405 |
| Time | - 0.029 | 0.041 | 0.515 | 0.473 | 0.971 |

Note: SE = Standard error, OR = Odds ratio.
