# Supplementary material for: Outcomes of the KC life 360 intervention: Improving employment and housing for persons living with HIV
Source: PLoS One. 2022 Sep 16;17(9):e0274923. doi: 10.1371/journal.pone.0274923 (PMC9481028; doi:10.1371/journal.pone.0274923)
Supplement: Attachment — Submitted filename: Response to Reviewers.docx [file pone.0274923.s007.docx]

| Comment | Response |
| --- | --- |
| Please ensure that your manuscript meets PLOS ONE's style requirements, including those for file naming. | We verified that the manuscript meets PLOS ONE’s style requirements. |
| Please include your full ethics statement in the ‘Methods’ section of your manuscript file. In your statement, please include the full name of the IRB or ethics committee who approved or waived your study, as well as whether or not you obtained informed written or verbal consent. If consent was waived for your study, please include this information in your statement as well. | We included that verbal consent was obtained. |
| Reviewer 1 |  |
| Generally, this is an important analysis because it describes the outcomes for a particularly vulnerable population in an underrepresented region of the US. However, the results are stated overly strongly and should be tempered by some discussion of how the lack of a comparison group limits generalizability. | We appreciate the reviewer for this comment. We tempered the language in the discussion and reported the lack of a comparison group as a limitation of this study. |
| Abstract: Are the % changes over baseline for all outcomes? Please include or describe in a standardized way. Please add the p values in the abstract. | We standardized the way we reported results in the abstract and included OR/p-values. |
| Introduction: Last paragraph in the introduction should outline more specifically what the relationships are under study in this analysis. | We included a hypothesis statement highlighting the specific relationship under study. |
| Methods: The sections included are not really standard and the reasons for the way they are ordered is a little unclear. Is recruitment the same as enrollment in the study? It should probably be grouped with data collection and measures, which are separate from the intervention description section. Suggest reordering. | We moved recruitment, participants, and data collection below the intervention characteristics. |
| Participants: The number listings in the participants paragraph are not needed. The definitions of homeless (literally, imminent, etc.) should be specified. | We removed the number listings. The definitions for housing status are listed in the measures, housing section. |
| Analysis: It looks like time is the proxy for the intervention because there’s no comparison group? This should be stated more clearly. | Time was not a proxy for the intervention. All participants received the intervention as described in the manuscript. Time in this case is an indicator of the length of time that an individual was exposed to the intervention. Time is an important predictor in this study because it represents the duration needed to achieve housing stability, employment, or viral suppression. |
| Results: The table names could be more specific, for example Table 1 might be better described as “Participant characteristics.” Table 2 should be “Frequency and percent of patients reporting outcomes” or something like that… | We changed the table names to be more appropriate. |
| The changes in clinical indicators might be better presented as figures in change over time? | We agree but PLOS publication guidelines only allow for 4 tables/figures. We feel that Tables 1 and 2 are very important and should be in the main body of the paper. If we were allowed to add additional figures, we would like to. |
| Are there statistical tests available across categories of outcomes? It looks like the supplemental tables have most of the statistical test, which should be in the paper instead. As an alternative, stat sig could be indicated in each category of housing in figure 1, for example. | We would like to include more of the tables in the paper. However, we want practitioners to use this information and we feel like they prefer simple figures versus tables. We include all of the supplemental tables so that researchers can view the details. PLOS guidelines do not allow us to have both tables and figures in the paper. We included stats in the notes below each figure. |
| Representing the results with a more traditional table/s of regression results would strengthen the paper. | We thank the reviewer for this comment. We would like to include additional tables that summarize the finding of the regression output in the main text. However, due to the table/figure limit, we cannot add additional tables to the main text. We include all results in the supplemental files and follow APA guidelines for regression output by reporting estimate, SE, p-value, and OR for all values. Because we have to include these results in supplemental tables, we feel that it’s best to report all of the statistics (e.g. ordinal intercept only, measurement wave, and measurement month) to be complete as possible. Traditional regression tables would not usually report this level of detail in main text tables. |
| Discussion The discussion argues that people achieved better outcomes than “before” the intervention, but there is no comparison data available for prior periods, just a baseline measure so the comparison is baseline to follow up, not really “before.” Similarly, is there any information on how many people achieve housing in a year, regardless of navigation? There must be some secular trend and the lack of a comparison group makes it difficult to assess how much of the change here is from that. Same goes for viral suppression so many of the findings are tentative without a comparison group and should be stated less strongly. The lack of a comparison group (contemporaneous or prior time period) is a key limitation and should be specified in the discussion. | We tempered the degree to which these results may be causal in the language of the discussion. We also noted the lack of a control group as a key limitation that should be the focus of future interventions and funding by federal agencies. |
| Reviewer 2: |  |
| This is an important study, and is well presented. Given that the study primarily addresses employment and housing, it would be useful for the authors to state whether the the navigation components include assistance with 'maintaining' housing and employment and the frequency of meetings with the navigators after housing and employment was obtained. Although included in the Discussion a 'Conclusion' section would be meaningful. | We thank the reviewer for this comment. We included additional information on maintaining housing and employment. PLOS guidelines do not allow for a conclusions section after the discussion. |
| Suggested Edits:  40: Add 'being' to reporting ... | Incorporated. |
| 50: Clarify whether gift cards were given 'during' or at completion of interview? | Incorporated in methods. |
| 65: All 'Americans' or to be more accurate, US residents or US population? | We changed this to be “The United States” |
| 68-69: Were meetings discontinued after clients were stably housed? | We incorporated, “Housing navigators continued to meet with clients as needed after achieving housing stability.” |
| 97: Consider deleting 'significantly'. | Deleted. |
| 135-136: Not sure if this is a standard definition; if not, please clarify "not having a job that used all their skills". | This is the standard definition that we have used for all studies on this project and that I used by HRSA. |
| 138-139: Recommend using a multilevel numbering system for the HIV health risk factors; e.g. lower case alphabets (a,b c) or roman numerals (i, ii, iii) if allowed. | Incorporated. |
| 158: Add documents to 'identification'. | Incorporated. |
| 183-185: Clarify - were case notes used to record current status/progress? | Yes, we clarified this in the text. |
| 183: Clarify whether documentation was on a shared system. | Clarified. |
| 186-187: What does local reporting mean i.e. who had access? | Only staff had access to the data. However, aggregate reports were generated to share results at local HIV service organizations, state and federal partners. We clarified this in the text. |
| 209, 211: Delete 'has' | Deleted. |
| 223: Consider changing to : 'was assessed at baseline to capture the last primary care visit ...' | Incorporated. |
| 250-252: Although listed under references, consider naming the scales used to assess depression and food insecurity. | We incorporated the names of these scales in the text. |
| 253: Consider listing all 12 unmet needs so that there is alignment with what you present in results. | We listed the additional unmet needs assessed. |
| 254: Change of to 'for' after need. | Incorporated. |
| 261-269: Change '<' symbols to commas? | Incorporated. |
| 309: Change 'were' to 'was'. | Incorporated. |
| 320: Previous sentence states viral suppression increased but sentence states retention in care 'also' decreased. This does not align. | We deleted “also.” |
| 323: Consider specifying 'change', i.e. replace with 'improved', 'increased', 'decreased', etc. | We replaced “change” with “improvements” to show direction. |
| 327: Change individual to individuals, i.e. singular to plural. | Incorporated. |
| 365: Consider replacing 'at' with 'related to'. | Incorporated. |
| 376: Add 'use of' client navigation to ... | Incorporated. |
| 379: Consider replacing 'compared to' with 'versus'. | Incorporated. |
| 383: Clarify direction of change. | We replaced “change” with “improve”. |
| 386: Change 'was to 'were'. | Incorporated. |
| 386-387: Confusing - please clarify. | We created two sentences from this run-on sentence to add clarification. |
| 392: Clarify - 'no differences' in variables or the impact of those variables? | Thank you for this comment as we believe this is a key point that needs to be considered in future research. We added language to clarify this for readers. |
| 400: Clarify - 'authors' or 'study'. | We replaced “the authors of this study” were replaced by the authors of the other studies. |
| Table 1: Clarify Food Security scores. In line 253 it is stated that higher scores indicate more food insecurity in which case it would be appropriate to title this as 'Food Insecurity'. | We changed this in Table 1. |
| Table 2: a) Explain discrepancy in numbers between various categories; b) Either include totals for each category or remove them. | A)We explained the discrepancy in sample sizes in the limitations section of the discussion. B) We removed the totals from the table. |
