# Supplementary material for: Outcomes of the KC life 360 intervention: Improving employment and housing for persons living with HIV
Source: PLoS One. 2022 Sep 16;17(9):e0274923. doi: 10.1371/journal.pone.0274923 (PMC9481028; doi:10.1371/journal.pone.0274923)
Supplement: Attachment — Submitted filename: Response to Reviewers#2.docx [file pone.0274923.s008.docx]

| Comment | Response |
| --- | --- |
| a) Please check for punctuation, spelling and grammar | We reviewed the manuscript for punctuation, spelling, and grammar. |
| b) Be specific when referring all PLWH vs study participants. | We changed all PLWH in the results section to “study participants” |
| 103: Delete 'to' | Deleted |
| 111: Replace 'be related to' with 'help promote' | Replaced |
| 121, 122: Delete 'who' | Deleted |
| 136: Replace 'received' with 'obtained' | Replaced |
| 142: Clarify 'emergency assistance' | Defined emergency assistance |
| 145: Combine 2nd sentence to preceding sentence stating 'and continued to to meet with them as needed after they achieved hosing stability'. | Changed |
| 150: Replace 'offices' with 'service sites' | Replaced |
| 175-181: Review punctuation and use of capital letters to help with clarity | Changed punctuation and use of capital letters to ensure clarity. |
| 200: Change capital 'T' to lower case in 'temporary' | Replaced |
| 219: Change to 'through lab data obtained from' | Changed |
| 224: Change 'were' to 'was' | Changed |
| 228: Change 'assessing' to 'assessed' | Changed |
| 229: add 'recorded' in | Included |
| 232: Add as 'being' out ... | Included |
| 269-276: Add '=' after each number e.g. (1)=literally ... | Included |
| 294: Add 'from study participants' | Included |
| 300: Replace 'were' to 'was' | Replaced |
| 307: Replace 'was' with 'were' | Replaced |
| 305, 307: Re-state 'average individual' to more accurately reflect results/analysis | Replaced “individual” with “participant” |
| 317: Change 'were' to 'was' | Replaced |
| 338: Change PLWH to 'study participants' | Changed throughout the results |
| 339: Change to - 'to have improved housing status' | Changed |
| 382: Clarify and specify - did a large proportion of study participants need additional support to get employment or to stay employed? | Thank you for this comment. Because we do not present dose of the intervention for employment, we removed this statement. |
| 387: 'Housing' or 'housing status'? | Included |
| 393: Context of? | Included “of the limitations” |
| 402: Delete extra period | Deleted |
| 403: attrition as well as retention? | Included “increase retention and decrease attrition..” |
| 407: Change to 'the study did not examine' or 'the results do not explain' | Changed |
| 426: Change data 'is' to 'are' | Changed |
| 427: Interviewed by whom? | Included “by study staff” |
| 429,430: Change to 'thus limiting' the ... | Changed |
| 431: Replace 'understand' with 'determine' | Replaced |
| 438: Should this be 'and scalability of' ... | Changed |
